# Socioeconomic inequalities in the uptake of postpartum care at home across Dutch neighbourhoods

**DOI:** 10.1093/eurpub/ckae089

**Published:** 2024-05-23

**Authors:** Leonie A Daalderop, Eline F de Vries, Eric A P Steegers, Jasper V Been, Jeroen N Struijs, Jacqueline Lagendijk

**Affiliations:** Department of Obstetrics and Gynaecology, Erasmus MC, University Medical Centre Rotterdam, Rotterdam, The Netherlands; Department of Quality of Care and Health Economics, Center of Prevention, Nutrition and Health Services Research, National Institute for Public Health and the Environment, Bilthoven, The Netherlands; Department of Obstetrics and Gynaecology, Erasmus MC, University Medical Centre Rotterdam, Rotterdam, The Netherlands; Department of Obstetrics and Gynaecology, Erasmus MC, University Medical Centre Rotterdam, Rotterdam, The Netherlands; Division of Neonatology, Department of Neonatal and Paediatric Intensive Care, Erasmus MC Sophia Children’s Hospital, University Medical Centre Rotterdam, Rotterdam, The Netherlands; Department of Public Health, Erasmus MC, University Medical Centre Rotterdam, Rotterdam, The Netherlands; Department of Quality of Care and Health Economics, Center of Prevention, Nutrition and Health Services Research, National Institute for Public Health and the Environment, Bilthoven, The Netherlands; Department of Public Health and Primary Care, Leiden University Medical Center, The Hague, The Netherlands; Department of Obstetrics and Gynaecology, Erasmus MC, University Medical Centre Rotterdam, Rotterdam, The Netherlands

## Abstract

**Background:**

Postpartum care focuses on prevention of health problems by performing medical check-ups and through enhancing maternal empowerment, the parent–infant interaction and knowledge about mother’s own health and that of her newborn. We aimed to investigate whether there was significant clustering within neighbourhoods regarding the uptake of postpartum care and to what extent neighbourhood-level differences are explained by individual socio-demographic factors, pregnancy-related factors and neighbourhood-level determinants (i.e. deprivation and urbanization).

**Methods:**

A nationwide population-based observational study was carried out using linked routinely collected healthcare data from appropriate-for-gestational-age weight live-born term singleton deliveries (2015–18) in the Netherlands. We performed two-level multivariable logistic regression analyses, using three different models. Model 1 contained no explanatory variables and was used to assess clustering of postpartum care uptake within neighbourhoods. In model 2, individual-level determinants were added one by one and in model 3, neighbourhood-level determinants were added.

**Results:**

About 520 818 births were included. Multilevel modelling showed that 11% of the total variance in postpartum care uptake could be attributed to the neighbourhood of residence. Individual characteristics explained 38% of the neighbourhood variance, of which income and migration background were the most important contributors. An additional 6% of the variation could be explained by neighbourhood-level determinants.

**Conclusion:**

We found substantial neighbourhood differences in postpartum care uptake. These differences are influenced by a complex interplay between individual-level and neighbourhood-level determinants, highlighting the importance of addressing both individual and neighbourhood-level determinants to improve the uptake of postpartum care and therewith overall community health.

## Introduction

The postpartum period—defined as the first six weeks after childbirth—is a physical, psychological and social transition phase for the mother and her family. Postpartum care focuses on prevention of health problems by performing medical check-ups and through enhancing maternal empowerment, the parent–infant interaction and knowledge about mother’s own health and that of her newborn.^1–3^ Additionally, postpartum care can facilitate the practice of breastfeeding,^4^ which can contribute to prolonged duration of breastfeeding.

The Netherlands has a unique postpartum care system, in which care is provided at home by skilled professionals in the first eight days after delivery. Although postpartum care is included in the basic insurance package, a co-payment is required for each hour of care (€4.30 per hour in 2018). The recommended total minimum volume of postpartum care at home is 24 h, the recommended volume is 49 h and the maximum amount is 80 h, depending on specific indications (see Supplementary file S1 for a detailed description of postpartum care allocation in the Netherlands). Postpartum care uptake below 24 h is more common among women with a low socioeconomic status (SES) or immigrant background.^5^ Additionally, these women and their offspring incur higher healthcare spending levels in the first year following delivery.^5^ Postpartum care may not only contribute to a possible reduction of healthcare spending in the first year after birth, but also reduces the incidence of low maternal empowerment.^1^ Enhanced maternal empowerment during the postpartum period in turn is associated with a range of positive health outcomes. By empowering women to take control of their health, societies can contribute to the well-being of mothers and the healthy development of the next generation. Doing so requires a clearer understanding of the factors contributing to inequalities in the uptake of postpartum care. So far, most research on the access to and uptake of antenatal and postpartum care focused on individual-level determinants such as income, marital status and education.^5^^,^^6–8^ However, it is increasingly recognized that neighbourhood-level determinants such as degree of urbanization or deprivation level also affect the health and healthcare utilization of residents.^9^^,^^10^ A neighbourhood is part of a municipality and often corresponds to a place of residence or part of a larger place of residence. In 2018, there were 3086 neighbourhoods in the Netherlands with an average area of 1060 hectares and 5567 residents (range 0–108 800). Individuals are known to cluster in neighbourhoods based on external constraints related to their SES and ethnicity.^11^ This spatial clustering of individual-level determinants may influence individual health-seeking behaviour through the presence of shared beliefs, social norms and attitudes towards care utilization, which are formed by social interactions.^12^^,^^13^ This idea is supported by the ‘social cohesion’ theory of Durkheim.^12^ Regarding the uptake of postpartum care it is unclear whether place of residence is an isolated risk factor or whether the risk is due to clustering of parents-to-be with similar socioeconomic risk profiles at the neighbourhood level.^11^ A clearer understanding of the role of the neighbourhood of residence on postpartum care uptake can help guide local interventions and policy plans to promote equality in the uptake of postpartum care.

Therefore, using multilevel modelling we aimed to investigate: (1) whether there was significant clustering within neighbourhoods regarding the uptake of postpartum care below the recommended minimum of 24 h and (2) to what extent neighbourhood-level differences in postpartum care uptake are explained by neighbourhood-level determinants and individual composition of the population.

## Methods

### Study design

Registered data at Perined of women living in the Netherlands who delivered a live-born term singleton baby between January 2015 and December 2018 were used to design a multilevel population-based observational study. Babies whose birth weight was below the 10th centile for gestational age and sex were excluded, as this is a common indication for prolonged hospitalization and therefore for reduced postpartum care at home. We assigned individuals (i.e. mothers and her child) to level 1, nested within neighbourhoods (i.e. maternal neighbourhood of residence in the year of childbirth) at level 2.

### Data sources

For this study, we used the DIAPER (Data-InfrAstructure of ParEnts and childRen) database, which contains individually linked data from three different national registries.^14^ Perinatal registry data were obtained from Perined, a Dutch organization that assembles statistical information on perinatal health. The Perined registry covers information on more than 97% of all pregnancies with a gestational age of 24 weeks and more (Perined, www.perined.nl). Routinely collected socio-demographic data were obtained from Statistics Netherlands. Lastly, claims data regarding postpartum care uptake were obtained from Vektis. Vektis is a database containing individual-level claims data from all Dutch health insurers covering more than 99% of the Dutch population. Maternity care organizations that provide postpartum care submit an invoice on behalf of the insured directly to the health insurer. Information regarding these claims are collected by Vektis. Statistics Netherlands provided a safeguarded platform where data were linked at the individual level using pseudonymized identification numbers (PINs). In order to link information of the mother and her child a pregnancy identification number was created. Detailed information regarding the linkage procedure can be found in Scheefhals et al.^14^

### Outcomes

The primary outcome was the uptake of postpartum care below the recommended minimum of 24 h of care. Hours of postpartum care were calculated by dividing the total postpartum care spending per person by the tariff that equal one hour of care, which differed per year. Postpartum care tariffs are set by the Dutch Healthcare Authority, and they do not differ between regions or organizations. We assumed that women did not make use of postpartum care if their registered postpartum care spending was lower than or equal to the tariff for the intake that is performed during pregnancy. These women were labelled as care below the recommended minimum. The proportion of women who did not make use of postpartum care was 3%. This percentage did not differ between 2015 and 2018.

### Exposure

#### Individual-level determinants

We took several socio-demographic and pregnancy-related factors into account that are known to be related to the uptake of postpartum care: disposable household income, highest completed educational qualification, home ownership, migration background, parenthood household status, parity and maternal age.^5^^,^^15^^,^^16^ Disposable household income was defined as the sum available from the household income for consumption and savings (i.e. net income) and categorized into low (<p20), moderate (p20–p80) and high (>p80). The highest completed educational qualification of the mother was based on the International Standard Classification of Education (ISCED) and divided into three categories: (1) low (pre-primary and primary education), (2) intermediate (education at the second level, first and second stage) and (3) high (education at the third level, first, second and third stage).^17^ Home ownership was dichotomized into owners–occupiers and no-owners (i.e. renters and others). Migration background of the mother was based on her parents' country of birth or her own country of birth and classified into non-immigrant, first generation and second generation. Parenthood household status was categorized into single parent household, two parent household and other, which includes institutionalized women and households that are not further specified. Parity was dichotomized into nulliparous and multiparous women. Maternal age was categorized into three age groups: <25; 25–35; >35 years.

#### Neighbourhood-level determinants

Neighbourhoods were defined using four-digit zip codes and municipal neighbourhood boundaries as recorded by Statistics Netherlands. We used the level of urbanization and neighbourhood deprivation as explanatory variables at the neighbourhood-level. The level of urbanization was based on address density per square kilometre (km^2^) and was categorized into urban (>1000 addresses per km^2^), moderate urban (500–1000 addresses per km^2^) and rural (<500 addresses per km^2^). Neighbourhood deprivation was based on the Neighbourhood Deprivation Index (NDI) formulated in 2012 by NIVEL, the Dutch Institute for Healthcare Research.^18^ The NDI is based on a composite score of the following items: (1) address density per square kilometre (km^2^), (2) percentage of non-Western immigrants, (3) percentage of households with a low income, (4) percentage of unemployed inhabitants. Deprivation was defined by NIVEL at an NDI of 4.9 or higher, rendering 224 neighbourhoods deprived (i.e. 884 355 people).

### Statistical analysis

#### Missing data

Both the individual and neighbourhood-level determinants were based on routinely collected data from Statistics Netherlands. Sixteen percent of the participants had a missing value in at least one of the individual or neighbourhood-level determinants. We performed multiple imputation using chained equations to account for this. Multiple predictor variables were used to inform the multiple imputation process, forming 10 datasets.

#### Multilevel analysis

First, maternal characteristics were compared between women who received postpartum care below versus equal to or above the minimum of 24 h. Subsequently, the prevalence of postpartum care below the recommended minimum of 24 h was determined for every neighbourhood using four-digit zip codes and municipal neighbourhood boundaries. The percentages of postpartum care below the recommended minimum of 24 h were illustrated on a map (figure 2). Thereafter, we performed two-level multivariable logistic regression analyses, using three different models. Model 1, the null model, contained no explanatory variables and was used to assess whether there is significant clustering of postpartum care uptake below the recommended minimum of 24 h within neighbourhoods. In model 2, the individual-level determinants were added one by one to assess which individual-level determinant explained most of the variance between neighbourhoods. This model was extended to form model 3, by adding the neighbourhood-level determinants one by one.

Associations between the individual-level determinants, neighbourhood-level determinants and the uptake of postpartum care were expressed as odds ratios. For each model, the random effects were expressed as the neighbourhood variance, proportional change in variance (PCV) and intraclass correlation coefficient (ICC). The neighbourhood variance represents each neighbourhood difference from the mean (i.e. postpartum care uptake <24 h), in which a higher variance reflects greater differences between neighbourhoods. The PCV expresses the proportion of the neighbourhood variance in model x that is explained by the added individual or neighbourhood-level determinants (see Formula 1).^19^
 

$$\text{PCV}=\frac{\left(\sigma^{2\text{model}\;x}-\sigma^{2\text{model}\;x+1}\right)}{\sigma^{2\text{model}\;x}}$$

 

***Formula 1.*** Proportional change in variance (PCV)

The ICC (see Formula 2) determines the proportion of variance in the outcome variable that is explained by differences between neighbourhoods.^20^ The ICC has a value between zero and one, in which a value below or equal to 0.10 is considered as little or no clustering, meaning a ‘small’ neighbourhood effect, a value between 0.10 and 0.24 is considered as a ‘medium’ neighbourhood effect and values equal to and greater than 0.25 are considered as a ‘large’ neighbourhood effect.^21^

$$\text{ICC}=\frac{\sigma^{2\;\text{neighbourhood}\;\text{model}\;x}}{\left(\sigma^{2\text{neighbourhood}\;\text{model}\;x}-\sigma^{2\text{individual}\;\text{model}\;x}\right)}$$

***Formula 2.*** Intraclass correlation coefficient (ICC)

In addition, several sensitivity analyses were performed. Consecutive pregnancies within the same mother have more characteristics in common than pregnancies between women. To investigate whether this dependency affected our findings, we excluded all consecutive pregnancies within the same mother and reran our analyses. To assess whether the imputed data affected our findings we performed a complete case analysis. Lastly, to check whether the women who did not take up any postpartum care biased our finding, we reran our analysis excluding these women. All statistical analyses were performed using Stata software (Stata SE 16.1, Stata Corporation, College Station, Texas, USA).

### Ethical approval

No formal ethical approval was needed for the analyses according to Dutch law, as the data used in this study are pseudonymized national registry data. Statistics Netherlands collects and produces population health statistics, referred to as non-public microdata, for all inhabitants of the Netherlands. Approval for the use of this microdata was obtained from the board of Statistics Netherlands, Perined and Vektis (project number 8099).

## Results

### Baseline characteristics

**Figure 1.**
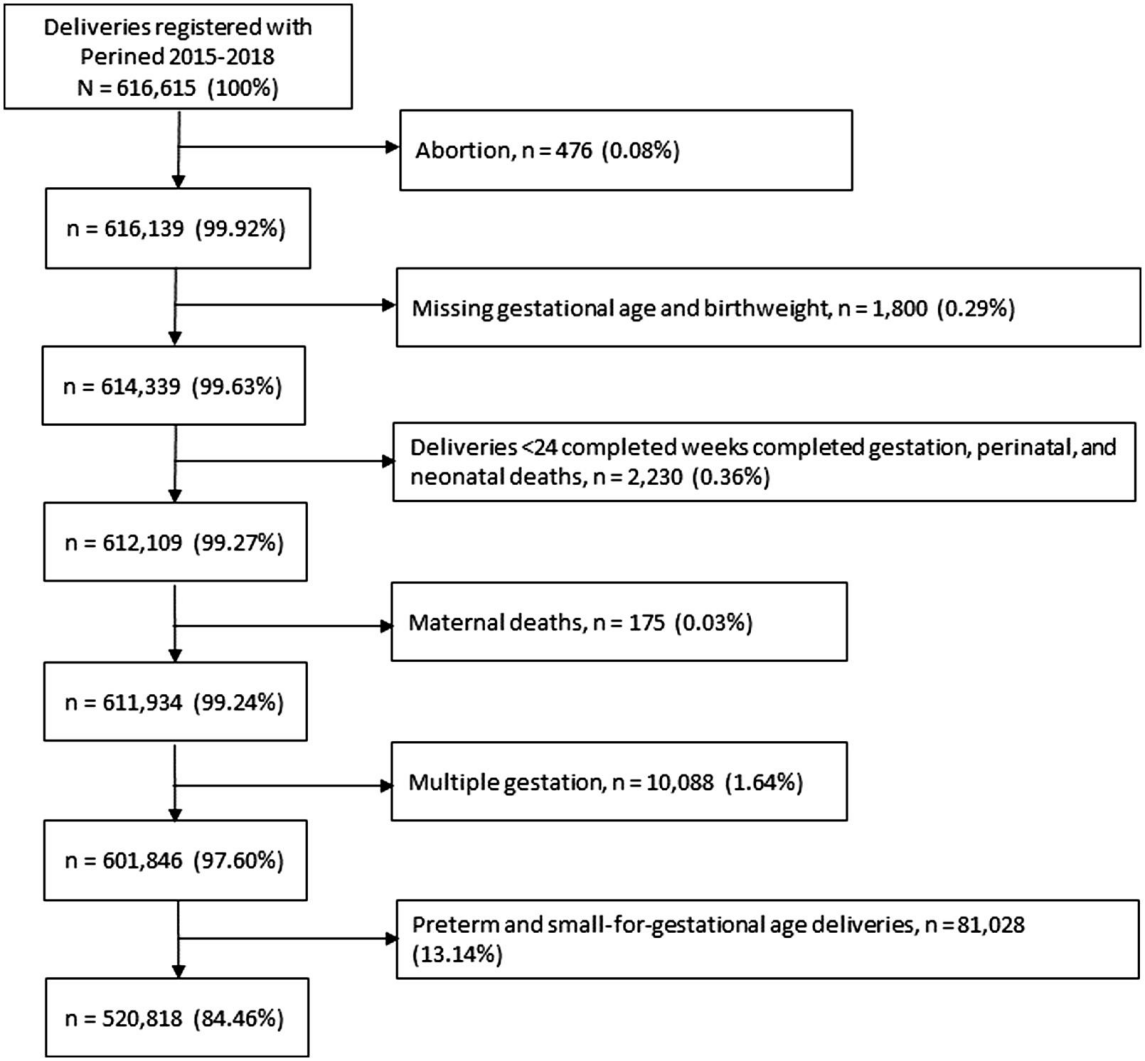
Flowchart

**Figure 2.**
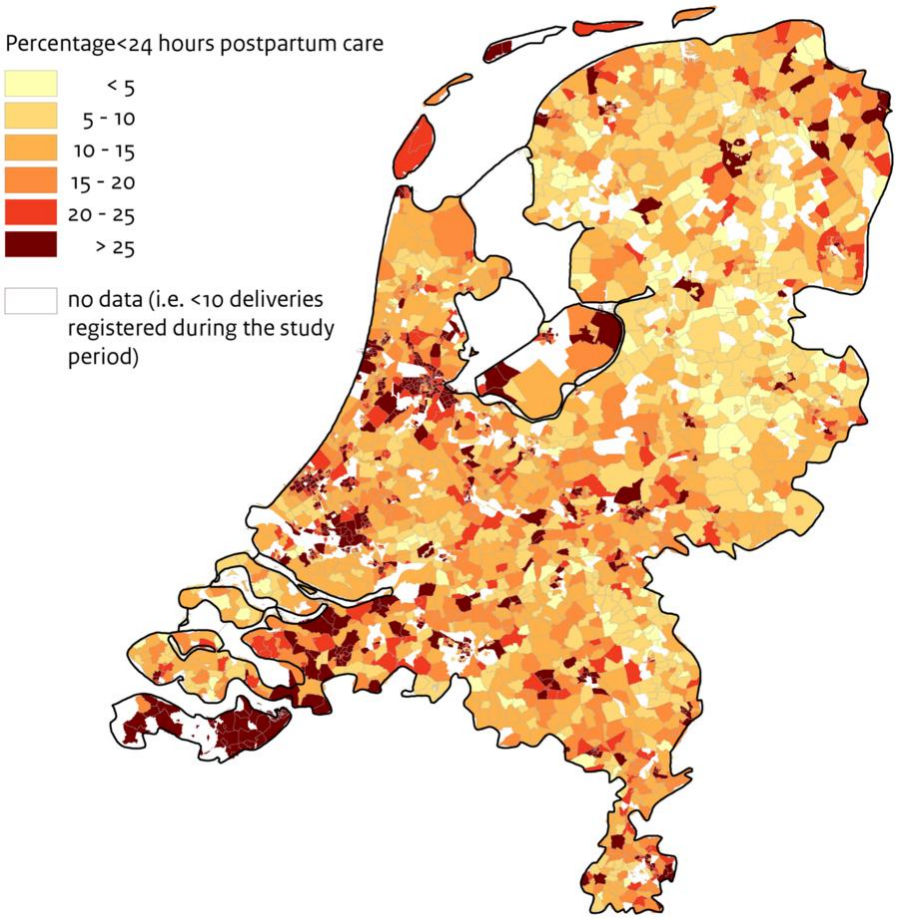
Spatial inequalities in postpartum care uptake <24 h

A total of 616 615 deliveries were registered with Perined between January 2015 and December 2018. Of these, we excluded preterm deliveries, small-for-gestational age deliveries and perinatal and neonatal deaths. Maternal deaths and multiple gestations were also excluded. In total, this study included 520 818 singleton appropriate-for-gestational-age weight live births to mothers who resided in 2741 neighbourhoods in the Netherlands (figure 1). The uptake of postpartum care was below the recommended minimum of 24 h in 20.4% of the women. At the neighbourhood level, postpartum care uptake below 24 h varied from 0% to as much as 83.3%. As shown in figure 2, postpartum care uptake below the recommended minimum was more common in big cities, such as Amsterdam, The Hague and Rotterdam. Women who received less than 24 h of postpartum care were more often born outside the Netherlands (41.1% vs. 12.1%) and single parents (20.1% vs. 9.2%) compared to women who received postpartum care equal to or above the recommended minimum. Additionally, all low-SES indicators were more prevalent among women who had a low uptake of postpartum care (table 1).

**Table 1:** Baseline characteristics

		Postpartum care uptake	
	Total (%)	≥24 h (%)	<24 h (%)
	*N* = 520 818 (100)	*N* = 414 399 (79.6)	*N* = 106 419 (20.4)
**Maternal characteristics**			
Maternal age (years)			
<25	47 153 (9.1)	29 937 (7.2)	17 216 (16.2)
25–35	363 841 (69.9)	296 765 (71.6)	67 076 (63.0)
>35	109 824 (21.1)	87 697 (21.2)	22 127 (20.8)
Parity			
Nulliparous	227 706 (43.7)	185 199 (44.7)	42 507 (39.9)
Multiparous	292 770 (56.2)	228 923 (55.2)	63 847 (60.0)
Missing	342 (0.1)	277 (0.1)	65 (0.1)
Migration background			
Non-immigrant	365 418 (70.2)	321 603 (77.6)	43 815 (41.2)
First generation	93 799 (18.0)	50 008 (12.1)	43 791 (41.1)
Second generation	61 601 (11.8)	42 788 (10.3)	18 813 (17.7)
Parenthood status			
Single parent	59 376 (11.4)	38 030 (9.2)	21 346 (20.1)
Two parents	447 721 (86.0)	368 574 (88.9)	79 147 (74.4)
Other	5228 (1.0)	2988 (0.7)	2240 (2.1)
Missing	8493 (1.6)	4807 (1.2)	3686 (3.5)
Urbanization			
Urban	295 963 (56.8)	223 029 (53.8)	72 934 (68.5)
Moderate urban	85 188 (16.4)	71 661 (17.3)	13 527 (12.7)
Rural	137 540 (26.4)	118 668 (28.6)	18 872 (17.7)
Missing	2127 (0.4)	1041 (0.3)	1086 (1.0)
**Socioeconomic characteristics**			
Educational level			
Lower education	20 587 (4.0)	8967 (2.2)	11 620 (10.9)
Intermediate education	226 107 (43.4)	175 315 (42.3)	50 792 (47.7)
Higher education	213 992 (41.1)	190 464 (46.0)	23 528 (22.1)
Missing	60 132 (11.5)	39 653 (9.6)	20 479 (19.2)
Disposable household income			
Low (<p20)	93 161 (17.9)	52 452 (12.7)	40 709 (38.3)
Moderate (p20-p80)	317 407 (60.9)	266 849 (64.4)	50 558 (47.5)
High (>p80)	104 145 (20.0)	92 247 (22.3)	11 898 (11.2)
Missing	6105 (1.2)	2851 (0.7)	3254 (3.1)
Home ownership			
Owner-occupiers	34 9427 (67.1)	306 089 (73.9)	43 338 (40.7)
No-owner (renters/others)	163 913 (31.5)	104 820 (25.3)	59 093 (55.5)
Missing	7478 (1.4)	3490 (0.8)	3988 (3.7)
Neighbourhood deprivation			
No	429 095 (82.4)	352 455 (85.1)	76 640 (72.0)
Yes	36 795 (7.1)	22 637 (5.5)	14 158 (13.3)
Missing	54 928 (10.5)	39 307 (9.5)	15 621 (14.7)

Values presented as frequencies and percentages.

### Neighbourhood of residence and postpartum care uptake

Multilevel modelling confirmed the differences between neighbourhoods regarding postpartum care uptake (table 2, model 1). In the null model, 11% of the total variance in postpartum care uptake could be attributed to the neighbourhood of residence. By including individual-level determinants in model 2, we observed that 38% of the neighbourhood variance in the null model could be explained by individual characteristics (table 2). Of the individual-level determinants, migration background (18%) and income (11%) were the largest contributors to the explained differences between neighbourhoods (Supplementary file S2). After adding the neighbourhood-level determinants in model 3, an additional 6% of the neighbourhood differences was explained (table 2, model 3). In total, the individual and neighbourhood-level determinants explained 44% of the differences in uptake of postpartum care between neighbourhoods. Findings were robust in sensitivity analyses, both in terms of neighbourhood variances, as well as direction and magnitude of the associations (Supplementary file S3).

**Table 2:** Multilevel associations between postpartum care uptake below the recommended minimum of 24 h and area and individual-level determinants

	**Model 1:** Null model	**Model 2:** Individual-level determinants added	**Model 3:** Neighbourhood-level determinants added
***Fixed effect, OR (95% CI)***			
**Individual-level determinants**			
Disposable household income			
Low <p20		1.74 (1.69–1.80)	1.75 (1.69–1.80)
Moderate p20–p80		1.12 (1.10–1.15)	1.13 (1.10-1.16)
High >p80		1.00^a^	1.00
Educational level			
Low		2.17 (2.08–2.26)	2.17 (2.08-2.27)
Intermediate		1.45 (1.42–1.48)	1.46 (1.43-1.49)
High		1.00	1.00
Home ownership			
Owner-occupiers		1.00	1.00
No-owner (renters/others)		1.68 (1.65–1.71)	1.66 (1.63-1.69)
Migration background			
Non-immigrant		1.00	1.00
First generation		3.55 (3.48–3.62)	3.50 (3.43-3.57)
Second generation		2.04 (2.00–2.09)	2.01 (1.96-2.05)
Parenthood status			
Single parent		1.17 (1.14–1.19)	1.16 (1.14-1.19)
Two parents		1.00	1.00
Other		1.60 (1.50–1.71)	1.59 (1.50-1.70)
Parity			
Nulliparous		1.00	1.00
Multiparous		1.22 (1.20–1.24)	1.22 (1.20-1.24)
Maternal age			
<25		1.69 (1.65–1.73)	1.69 (1.65-1.73)
25–35		1.00	1.00
>35		0.98 (0.97–1.00)	0.98 (0.96-1.00)
**Neighbourhood-level determinants**			
Urbanization			
Urban			1.00
Moderate urban			0.79 (0.76-0.83)
Rural			0.74 (0.71-0.77)
Neighbourhood deprivation			
No			1.00
Yes			1.12 (1.08-1.15)
***Random effect***			
Neighbourhood variance (SE)	0.62 (SE 0.01)	0.38 (SE 0.01)	0.35 (SE 0.01)
Proportional change in variance	Reference	0.39	0.44
Intraclass correlation	0.11	0.04	0.04

OR, odds ratio; SE, standard error.

a OR of 1.00 is meaning that the displayed category is the reference category.

## Discussion

With this population-based observational study using national linked data of over half a million singleton pregnancies, we found substantial neighbourhood differences in postpartum care uptake. In some neighbourhoods, the uptake of postpartum care below the recommended minimum amount was four times higher (up to 83.3%) than the national average of 20.4%. This lack in uptake and the differences between neighbourhoods were most obvious in the big cities, such as Amsterdam, The Hague and Rotterdam. This study shows that 11% of the total variance in postpartum care uptake can be attributed to the neighbourhood of residence. Variation in the uptake of postpartum care between neighbourhoods was predominantly explained by individual characteristics (38%), of which migration background and income explained most of the variance. An additional 6% of the variation could be explained by neighbourhood-level determinants, leaving 56% unexplained.

Strengths of this study include the use of a nationally representative database with claims data and socio-demographic data linked at the individual level. Application of the multilevel design helped disentangle the contribution of individual and neighbourhood-level determinants to differences in postpartum care uptake between neighbourhoods. When interpreting our results, several limitations merit discussion. First, some of the national registries from Statistics Netherlands had missing values, with the highest proportion missing values for educational qualification (11.5%). To minimize bias caused by the missing values we performed multiple imputation. Sensitivity analyses on complete cases yielded similar results compared to the analysis on imputed data, supporting validity of the imputation and our findings. Second, the uptake of postpartum care was provided in total expense rather than total received hours, making derivation necessary. This might have led to misclassification of the received postpartum care hours. Third, postpartum care is only delivered in the first eight days following delivery. Therefore, prolonged hospitalization after delivery increases the chance of uptake of postpartum care below the recommended minimum of 24 h. Unfortunately, we were not able to remove all persons with prolonged hospitalization as our dataset lacked information on the days between delivery and start of postpartum care. Yet, we did exclude all multiple gestations, preterm births and small-for-gestational age deliveries as these are a main causes of prolonged hospitalization after delivery. Fourth, the design of our analyses assumes that the magnitude of the association between the individual-level determinants and postpartum care uptake is similar in all neighbourhoods. However, it is possible that the neighbourhood context affects specific groups (e.g. low income, specific ethnic groups) differently. Fifth, we only had dichotomized data available regarding neighbourhood deprivation. This makes it impossible to study an exposure−response relation between degree of neighbourhood deprivation and the uptake of postpartum care. Future research is necessary to elucidate the existence of a specific exposure−response relation. Lastly, address density was used as input at two levels; for both the determinants neighbourhood deprivation and urbanization level, albeit in distinct ways. Theoretically this may have led to a small degree of overcorrection.

Our research aligns with previous studies, demonstrating a consistent inequality in the distribution of primary healthcare in which socioeconomically disadvantaged groups receive less care.^5^^,^^22^^,^^23^ The association between socioeconomic disadvantage and healthcare utilization is well-established and multifaceted.^24–27^ Besides the problem that there is a consistent inequality in postpartum care provision, there is also a growing shortage of skilled professionals who can provide postpartum care in the Netherlands. To safeguard the unique character of postpartum care in the Netherlands, it may well be important to prioritize care to those in greatest need. The findings from this study can contribute to a more equitable postpartum care distribution. We demonstrate that it is important to take income and migration background of parents-to-be into account in the allocation of postpartum care. Low-income individuals often face significant barriers that impact their ability to access and utilize healthcare services effectively. Some key aspects of this association include lower health literacy^28^^,^^29^ and the co-payment that is required for each hour of postpartum care. A previous qualitative study^15^ among vulnerable women in the Netherlands showed that the required co-payment resulted in lower postpartum care uptake among several of the interviewees. When striving for a more equitable postpartum care distribution, policy-makers and health insurers should consider waiving the co-payment, particularly among vulnerable women. Future research should investigate whether waiving this co-payment may be helpful in increasing the uptake of postpartum care among disadvantaged women.

In line with previous work, we found that migration background was an important individual-level determinant that explained most of the neighbourhood variance in postpartum care uptake.^5^ Women born outside the Netherlands were less likely to utilize postpartum care equal to or above the recommended minimum of 24 h. This may be due to cultural differences, including relying more on care provided through personal social networks. Underutilization of care among ethnic minorities is however also observed in antenatal healthcare services.^30–32^ Although we only had data available on first and second generation migrants, it is also important to study the uptake of postpartum care among asylum seekers/refugees and labour migrants. This is an extremely vulnerable group that experience an increased rate of adverse pregnancy outcomes compared to native women.^33^^,^^34^ Lack of knowledge of or information about the antenatal healthcare system, poor language proficiency and cultural and religious beliefs are reported barriers for antenatal healthcare utilization among ethnic minorities.^35^ Addressing the impact of migration background on healthcare utilization requires efforts to improve cultural competence among healthcare providers, provide language assistance services and develop outreach programs targeting migrant communities, asylum seekers/refugees and labour migrants.

Forty-four percent of the variance between neighbourhoods could be explained by the investigated individual and neighbourhood-level determinants, leaving 56% unexplained. In addition to the factors we investigated, other individual and neighbourhood-level determinants also play a role in the uptake of postpartum care. Possible individual-level determinants that influence the uptake of care are for example limited health literacy, health beliefs, and distrust of the healthcare system.^36^^,^^37^ Limited health literacy can affect the understanding of healthcare services and the importance of preventive care such as postpartum care. Personal health beliefs and priorities can influence whether individuals prioritize preventive healthcare including postpartum care. Additionally, disadvantaged populations often experience preventive care as ‘interference care’ which may affect their uptake of care. Distrust of the healthcare system and negative prior experiences contribute to this. Qualitative research methods such as interviews and focus groups can help determine whether the above factors influence the uptake in postpartum care and how to address these factors. This enables the development of policy plans and targeted interventions to achieve a more equitable postpartum care system.

Neighbourhood-level determinants that may have an impact on the uptake of postpartum care are the existence of a strong community and local policies and initiatives to promote the uptake of preventive healthcare. A strong community and social support systems can motivate individuals to engage in preventive healthcare. Social norms and peer influence play a significant role in encouraging healthy behaviours.^12^^,^^13^

The results described above and our consideration of them underlines the need for a more equal distribution of postpartum care. Managers of postpartum care organizations should recognize this necessity more. Given the persistent health disparities based on someone’s SES,^38^ we believe it is time to make equality in health care use a priority. This may include postpartum care allocation based on SES-related factors. A first step towards a better allocation of care hours will have to be arranged at the neighbourhood level. Maternity care organizations should adapt their protocols of allocation care to the regional situation.

To conclude, our study found that the uptake of postpartum care is influenced by a complex interplay between individual-level and neighbourhood-level determinants. The interplay between these factors highlights the importance of addressing both individual and neighbourhood-level determinants to improve the uptake of postpartum care and therewith overall community health. Efforts to enhance the uptake of postpartum care should therefore include educating and motivating individuals but also creating supportive environments that facilitate this.

## Supplementary Material

ckae089_Supplementary_Data

## Data Availability

The data underlying this article were provided by Statistics Netherlands, Perined and Vektis by permission. Data will be shared on request to the corresponding author with permission of Statistics Netherlands, Perined and Vektis. Key points Multilevel modelling showed differences between neighbourhoods in the degree to which women make use of postpartum care. The uptake of postpartum care below the recommended minimum of 24 h is determined by a complex interplay between individual-level and neighbourhood-level determinants. Having a low income or a migration background were the key determinants of reduced uptake of postpartum care. Efforts to enhance the uptake of postpartum care should include educating and motivating individuals but also creating supportive environments that facilitate this.

## References

[1] Lagendijk J , Sijpkens MK, Ernst-Smelt HE, et al Risk-guided maternity care to enhance maternal empowerment postpartum: a cluster randomized controlled trial. PLoS One 2020;15:e0242187. 33216791 10.1371/journal.pone.0242187 PMC7679010

[2] Shaw E , Levitt C, Wong S, Kaczorowski J. Systematic review of the literature on postpartum care: effectiveness of postpartum support to improve maternal parenting, mental health, quality of life, and physical health. Birth 2006;33:210–20. 16948721 10.1111/j.1523-536X.2006.00106.x

[3] Mokhtari F , Bahadoran P, Baghersad Z. Effectiveness of postpartum homecare program as a new method on mothers’ knowledge about the health of the mother and the infant. Iran J Nurs Midwifery Res 2018;23:316–21. 30034494 10.4103/Ijnmr.ijnmr_48_17 PMC6034528

[4] Tiruneh GT , Shiferaw CB, Worku A. Effectiveness and cost-effectiveness of home-based postpartum care on neonatal mortality and exclusive breastfeeding practice in low-and-middle-income countries: a systematic review and meta-analysis. BMC Pregnancy Childbirth 2019;19:507–19. 31852432 10.1186/s12884-019-2651-6 PMC6921506

[5] Lagendijk J , Steegers EAP, Been JV. Inequity in postpartum healthcare provision at home and its association with subsequent healthcare expenditure. Eur J Public Health 2019;29:849–55. 31329862 10.1093/eurpub/ckz076 PMC6761843

[6] Blondel B , Marshall B. Poor antenatal care in 20 French districts: risk factors and pregnancy outcome. J Epidemiol Community Health 1998;52:501–6. 9876361 10.1136/jech.52.8.501 PMC1756747

[7] Delvaux T , Buekens P, Godin I, Boutsen M. Barriers to prenatal care in Europe. Am J Prev Med 2001;21:52–9. 11418258 10.1016/s0749-3797(01)00315-4

[8] Partridge S , Balayla J, Holcroft CA, Abenhaim HA. Inadequate prenatal care utilization and risks of infant mortality and poor birth outcome: a retrospective analysis of 28,729,765 US deliveries over 8 years. Am J Perinatol 2012;29:787–93. 22836820 10.1055/s-0032-1316439

[9] Diez Roux AV. Investigating neighborhood and area effects on health. Am J Public Health 2001;91:1783–9. 11684601 10.2105/ajph.91.11.1783 PMC1446876

[10] Kawachi I , Berkman LF. Neighborhoods and Health. New York: Oxford University Press, 2003.

[11] Diez Roux AV , Mair C. Neighborhoods and health. Ann N Y Acad Sci 2010;1186:125–45. 20201871 10.1111/j.1749-6632.2009.05333.x

[12] Kawachi I , Berkman L. Social cohesion, social capital, and health. In: Kawachi I, Berkman L, editors. Social Epidemiology. New York: Oxford University Press, 2000: 290–319.

[13] Mohnen SM , Schneider S, Droomers M. Neighborhood characteristics as determinants of healthcare utilization – a theoretical model. Health Econ Rec 2019;9:1–9. 10.1186/s13561-019-0226-x PMC6734422 30840211

[14] Scheefhals ZTM , de Vries EF, Molenaar JM, et al Observational data for integrated maternity care: experiences with a data-infrastructure for parents and children in the Netherlands. Int J Integr Care 2023;23:20. 10.5334/ijic.7012 PMC10742107 38145057

[15] Laureij LT , Van Der Hulst M, Lagendijk J, et al Insight into the process of postpartum care utilisation and in-home support among vulnerable women in the Netherlands: an in-depth qualitative exploration. BMJ Open 2021;11:e046696. 10.1136/bmjopen-2020-046696 PMC8422309 34489272

[16] Adane B , Fisseha G, Walle G, Yalew M. Factors associated with postnatal care utilization among postpartum women in Ethiopia: a multi-level analysis of the 2016 Ethiopia demographic and health survey. Arch Pub Health 2020;78:1–10. 32322394 10.1186/s13690-020-00415-0 PMC7161122

[17] Schaart R , Mies MB, Westerman S. *The Dutch Standard Classification of Education, SOI 2006*. Statistics Netherlands; 2008.

[18] Devillé W , Wiegers T. *Herijking stedelijke achterstandsgebieden 2012*. NIVEL; 2012.

[19] Merlo J , Yang M, Chaix B, et al A brief conceptual tutorial on multilevel analysis in social epidemiology: investigating contextual phenomena in different groups of people. J Epidemiol Community Health 2005;59:729–36. 16100308 10.1136/jech.2004.023929 PMC1733145

[20] Merlo J , Chaix B, Yang M, et al A brief conceptual tutorial of multilevel analysis in social epidemiology: linking the statistical concept of clustering to the idea of contextual phenomenon. J Epidemiol Community Health 2005;59:443–9. 15911637 10.1136/jech.2004.023473 PMC1757045

[21] LeBreton JM , Senter JL. Answers to 20 questions about interrater reliability and interrater agreement. Organ Res Methods 2008;11:815–52.

[22] Ahmed S , Creanga AA, Gillespie DG, Tsui AO. Economic status, education and empowerment: implications for maternal health service utilization in developing countries. PLoS One 2010;5:e11190. 20585646 10.1371/journal.pone.0011190 PMC2890410

[23] Lindquist A , Kurinczuk JJ, Redshaw M, Knight M. Experiences, utilisation and outcomes of maternity care in England among women from different socio‐economic groups: findings from the 2010 National Maternity Survey. BJOG 2015;122:1610–7. 25227878 10.1111/1471-0528.13059

[24] Vikum E , Bjørngaard JH, Westin S, Krokstad S. Socio-economic inequalities in Norwegian health care utilization over 3 decades: the HUNT Study. Eur J Public Health 2013;23:1003–10. 23729479 10.1093/eurpub/ckt053

[25] Sortsø C , Lauridsen J, Emneus M, et al Socioeconomic inequality of diabetes patients’ health care utilization in Denmark. Health Econ Rev 2017;7:21–2. 28550486 10.1186/s13561-017-0155-5 PMC5446432

[26] Okoli C , Hajizadeh M, Rahman MM, Khanam R. Geographical and socioeconomic inequalities in the utilization of maternal healthcare services in Nigeria: 2003–2017. BMC Health Serv Res 2020;20:849–14. 32912213 10.1186/s12913-020-05700-w PMC7488161

[27] Lueckmann SL , Hoebel J, Roick J, et al Socioeconomic inequalities in primary-care and specialist physician visits: a systematic review. Int J Equity Health 2021;20:58–19. 33568126 10.1186/s12939-020-01375-1 PMC7874661

[28] Protheroe J , Whittle R, Bartlam B, et al Health literacy, associated lifestyle and demographic factors in adult population of an English city: a cross‐sectional survey. Health Expect 2017;20:112–9. 26774107 10.1111/hex.12440 PMC5217902

[29] Sørensen K , Pelikan JM, Röthlin F, et al Health literacy in Europe: comparative results of the European health literacy survey (HLS-EU). Eur J Public Health 2015;25:1053–8. 25843827 10.1093/eurpub/ckv043 PMC4668324

[30] Alderliesten ME , Vrijkotte TGM, Van Der Wal MF, Bonsel GJ. Late start of antenatal care among ethnic minorities in a large cohort of pregnant women. BJOG 2007;114:1232–9. 17655734 10.1111/j.1471-0528.2007.01438.x

[31] Boerleider AW , Manniën J, van Stenus CMV, et al Explanatory factors for first and second-generation non-western women’s inadequate prenatal care utilisation: a prospective cohort study. BMC Pregnancy Childbirth 2015;15:98–10. 25895975 10.1186/s12884-015-0528-x PMC4409999

[32] Choté AA , De Groot CJ, Bruijnzeels MA, et al Ethnic differences in antenatal care use in a large multi-ethnic urban population in the Netherlands. Midwifery 2011;27:36–41. 19939527 10.1016/j.midw.2009.07.008

[33] Tankink JB , Verschuuren AEH, Postma IR, et al Childbirths and the prevalence of potential risk factors for adverse perinatal outcomes among asylum seekers in the Netherlands: a five-year cross-sectional study. Int J Environ Res Public Health 2021;18:12933. 34948540 10.3390/ijerph182412933 PMC8700803

[34] van Hanegem N , Solnes Miltenburg A, Zwart JJ, et al Severe acute maternal morbidity in asylum seekers: a two-year nationwide cohort study in the Netherlands. Acta Obstet Gynecol Scand 2011;90:1010–6. 21446931 10.1111/j.1600-0412.2011.01140.x

[35] Boerleider AW , Wiegers TA, Manniën J, et al Factors affecting the use of prenatal care by non-western women in industrialized western countries: a systematic review. BMC Pregnancy Childbirth 2013;13:81–11. 23537172 10.1186/1471-2393-13-81 PMC3626532

[36] Scott TL , Gazmararian JA, Williams MV, Baker DW. Health literacy and preventive health care use among Medicare enrollees in a managed care organization. Med Care 2002;40:395–404. 11961474 10.1097/00005650-200205000-00005

[37] Chen X , Hay JL, Waters EA, et al Health literacy and use and trust in health information. J Health Commun 2018;23:724–34. 30160641 10.1080/10810730.2018.1511658 PMC6295319

[38] Bertens LCM , Burgos Ochoa L, van Ourti T, et al Persisting inequalities in birth outcomes related to neighbourhood deprivation. J Epidemiol Community Health 2020;74:232–9. 31685540 10.1136/jech-2019-213162 PMC7035720

