# Supplementary material for: Socioeconomic inequalities in the uptake of postpartum care at home across Dutch neighbourhoods
Source: Eur J Public Health. 2024 May 23;34(5):921–8. doi: 10.1093/eurpub/ckae089 (PMC11430907; doi:10.1093/eurpub/ckae089)
Supplement: ckae089_Supplementary_Data [file ckae089_supplementary_data.pdf]

Supplementary file 1. Postpartum care allocation in the Netherlands

The Netherlands has a unique perinatal health care system, which is based on the principle that pregnancy, childbirth, and the postpartum period are fundamentally physiological processes (1). At the primary care level, low-risk pregnant women are guided and supported by community midwives (2, 3). If complications occur, or threaten to occur, pregnant women are referred to hospital-based obstetric care provided by obstetricians (secondary and tertiary level of care) . Low-risk pregnant women can opt for an out-of-hospital delivery either at home, in a primary care birth centre, or in an outpatient clinic. After delivery, these women are offered postpartum care at home provided by maternity care assistants (MCAs), who are being supervised by community midwives (4). The purpose of postpartum care is to promote the physical, social, and mental health of mother and child and to detect complications at an early stage. Moreover, MCAs help parents to cope with their new situation, by supporting them in taking care of their newborn and enhancing their empowerment (4).

Postpartum care is usually delivered during the first eight days after delivery, for six to eight hours a day, tapering to fewer hours per days towards the end of the care period. During pregnancy a MCA will assess a woman's expected care requirements, based on the indications denoted in the Dutch national indication protocol (abbreviation 'LIP' in Dutch), during an intake appointment between 25 and 37 weeks of gestation. Examples of indications that add to the intensity of care are: not being physically self-sufficient, having a psychological illness, and having other children under the age of four. The minimum volume of postpartum care at home is 24 hours, the recommended volume 49 hours, and the maximum amount 80 hours, depending on specific indications. Postpartum care is covered by the general health care insurance (which is mandatory for every Dutch inhabitant) with exception of an out-of-pocket payment of 4.30 per hour in 2018 .

References

1. Verloskundig vademecum 2003. Eindrapport van de Commissie Verloskunde van het College voor zorgverzekeringen. College voor zorgverzekeringen, Diemen; 2003. Report No.: 90-70918-34-X
2. Amelink-Verburg MP, Buitendijk SE. Pregnancy and labour in the Dutch maternity care system: what is normal? The role division between midwives and obstetricians. J Midwifery Womens Health 2010;55(3):216-25.
3. Bais JMJ, Pel M. The basis of the Dutch obstetric system: risk selection. European Clinics in Obstetrics and Gynaecology 2006;2(4):209-12.

4. Wiegers TA. Adjusting to motherhood: maternity care assistance during the postpartum period: how to help new mothers cope. Journal of Neonatal Nursing 2006;12(5):163-71.

Supplementary file 2. Multilevel associations between postpartum care uptake below the recommended minimum of 24 hours, individual and neighbourhood level determinants added one by one

Table S1. Multilevel associations with individual determinant income added

	**Model 1:** Null model	**Model 2:** Individual level determinants added
Fixed effect, OR (95%-CI)		
**Individual level determinants**		
Disposable income		
Low <p20		5.36 (5.22-5.49)
Moderate p20-p80		1.53 (1.50-1.57)
High >p80		1.00
Random effect		
Neighbourhood variance (SE)	0.62 (SE 0.01)	0.55 (SE 0.01)
Proportional change in variance	Reference	0.11
Intraclass correlation	0.11	0.09

Abbreviations: OR: odds ratio, SE: standard error
*OR of 1.00 is meaning that the displayed category is the reference category

Table S2. Multilevel associations with individual determinants income and education added

	**Model 1:** Null model	**Model 2:** Individual level determinants added
Fixed effect, OR (95%-CI)		
**Individual level determinants**		
Disposable income		
Low <p20		3.43 (3.33-3.52)
Moderate p20-p80		1.29 (1.26-1.32)
High >p80		1.00*
Educational level		
Low		4.06 (3.90-4.23)
Intermediate		1.70 (1.67-1.74)
High		1.00
Random effect		
Neighbourhood variance (SE)	0.62 (SE 0.01)	0.54 (SE 0.01)
Proportional change in variance	Reference	0.13
Intraclass correlation	0.11	0.08

Abbreviations: OR: odds ratio, SE: standard error
*OR of 1.00 is meaning that the displayed category is the reference category

Table S3. Multilevel associations with individual determinants income, education, and home ownership added

	**Model 1:** Null model	**Model 2:** Individual level determinants added
Fixed effect, OR (95%-CI)		
**Individual level determinants**		
Disposable income		
Low <p20		2.29 (2.23-2.36)
Moderate p20-p80		1.19 (1.16-1.21)
High >p80		1.00*
Educational level		
Low		3.46 (3.32-3.60)
Intermediate		1.58 (1.55-1.61)
High		1.00
Home ownership		
Owner-occupiers		1.00
No-owner (renters/others)		2.07 (2.04-2.11)
Random effect		
Neighbourhood variance (SE)	0.62 (SE 0.01)	0.49 (SE 0.01)
Proportional change in variance	Reference	0.21
Intraclass correlation	0.11	0.07

Abbreviations: OR: odds ratio, SE: standard error
*OR of 1.00 is meaning that the displayed category is the reference category

Table S4. Multilevel associations with individual determinants income, education, home ownership, and migration background added

	**Model 1:** Null model	**Model 2:** Individual level determinants added
Fixed effect, OR (95%-CI)		
**Individual level determinants**		
Disposable income		
Low <p20		2.00 (1.94-2.06)
Moderate p20-p80		1.17 (1.15-1.20)
High >p80		1.00*
Educational level		
Low		2.32 (2.22-2.42)
Intermediate		1.53 (1.50-1.57)
High		1.00
Home ownership		
Owner-occupiers		1.00
No-owner (renters/others)		1.74 (1.71-1.78)
Migration background		
Non-immigrant		1.00
First generation		3.44 (3.37-3.51)
Second generation		2.06 (2.02-2.11)
Random effect		
Neighbourhood variance (SE)	0.62 (SE 0.01)	0.38 (SE 0.01)
Proportional change in variance	Reference	0.39
Intraclass correlation	0.11	0.04

Abbreviations: OR: odds ratio, SE: standard error

*OR of 1.00 is meaning that the displayed category is the reference category

Table S5. Multilevel associations with individual determinants income, education, home ownership, migration background, and parenthood status added

	**Model 1:** Null model	**Model 2:** Individual level determinants added
Fixed effect, OR (95%-CI)		
**Individual level determinants**		
Disposable income		
Low <p20		1.93 (1.87-1.99)
Moderate p20-p80		1.17 (1.14-1.20)
High >p80		1.00*
Educational level		
Low		2.31 (2.21-2.40)
Intermediate		1.53 (1.49-1.56)
High		1.00
Home ownership		
Owner-occupiers		1.00
No-owner (renters/others)		1.70 (1.67-1.74)
Migration background		
Non-immigrant		1.00
First generation		3.46 (3.39-3.53)
Second generation		2.05 (2.01-2.10)
Parenthood status		
Single parent		1.15 (1.13-1.18)
Two parents		1.00
Other		1.66 (1.56-1.76)
Random effect		
Neighbourhood variance (SE)	0.62 (SE 0.01)	0.38 (SE 0.01)
Proportional change in variance	Reference	0.39
Intraclass correlation	0.11	0.04

Abbreviations: OR: odds ratio, SE: standard error
*OR of 1.00 is meaning that the displayed category is the reference category

Table S6. Multilevel associations with individual determinants income, education, home ownership, migration background, parenthood status, and parity added

	**Model 1:** Null model	**Model 2:** Individual level determinants added
Fixed effect, OR (95%-CI)		
**Individual level determinants**		
Disposable income		
Low <p20		1.84 (1.78-1.90)
Moderate p20-p80		1.14 (1.12-1.17)
High >p80		1.00*
Educational level		
Low		2.29 (2.19-2.38)
Intermediate		1.53 (1.50-1.56)
High		1.00
Home ownership		
Owner-occupiers		1.00
No-owner (renters/others)		1.73 (1.70-1.76)
Migration background		
Non-immigrant		1.00
First generation		3.45 (3.38-3.52)
Second generation		2.05 (2.00-2.10)
Parenthood status		
Single parent		1.17 (1.15-1.20)
Two parents		1.00
Other		1.70 (1.60-1.81)
Parity		
Nulliparous		1.00
Multiparous		1.12 (1.10-1.14)
Random effect		
Neighbourhood variance (SE)	0.62 (SE 0.01)	0.38 (SE 0.01)
Proportional change in variance	Reference	0.39
Intraclass correlation	0.11	0.04

Abbreviations: OR: odds ratio, SE: standard error

*OR of 1.00 is meaning that the displayed category is the reference category

Table S7. Multilevel associations with individual determinants income, education, home ownership, migration background, parenthood status, parity, and maternal age added

	**Model 1:**<br>Null model	**Model 2:**<br>Individual level determinants added
Fixed effect, OR (95%-CI)		
**Individual level determinants**		
Disposable income		
Low <p20		1.74 (1.69-1.80)
Moderate p20-p80		1.12 (1.10-1.15)
High >p80		1.00*
Educational level		
Low		2.17 (2.08-2.26)
Intermediate		1.45 (1.42-1.48)
High		1.00
Home ownership		
Owner-occupiers		1.00
No-owner (renters/others)		1.68 (1.65-1.71)
Migration background		
Non-immigrant		1.00
First generation		3.55 (3.48-3.62)
Second generation		2.04 (2.00-2.09)
Parenthood status		
Single parent		1.17 (1.14-1.19)
Two parents		1.00
Other		1.60 (1.50-1.71)
Parity		
Nulliparous		1.00
Multiparous		1.22 (1.20-1.24)
Maternal age		
<25		1.69 (1.65-1.73)
25-35		1.00
>35		0.98 (0.97-1.00)
Random effect		
Neighbourhood variance (SE)	0.62 (SE 0.01)	0.38 (SE 0.01)
Proportional change in variance	Reference	0.39
Intraclass correlation	0.11	0.04

Abbreviations: OR: odds ratio, SE: standard error
*OR of 1.00 is meaning that the displayed category is the reference category

Table S8. Multilevel associations with individual determinants income, education, home ownership, migration background, parenthood household status, parity, and neighbourhood level determinant urbanisation

	**Model 1:** Null model	**Model 2:** Individual level determinants added	**Model 3:** Neighbourhood level determinants added
Fixed effect, OR (95%-CI)			
**Individual level determinants**			
Disposable income			
Low <p20		1.74 (1.69-1.80)	1.75 (1.70-1.81)
Moderate p20-p80		1.12 (1.10-1.15)	1.13 (1.10-1.16)
High >p80		1.00*	1.00
Educational level			
Low		2.17 (2.08-2.26)	2.18 (2.09-2.27)
Intermediate		1.45 (1.42-1.48)	1.46 (1.43-1.49)
High		1.00	1.00
Home ownership			
Owner-occupiers		1.00	1.00
No-owner (renters/others)		1.68 (1.65-1.71)	1.67 (1.64-1.70)
Migration background			
Non-immigrant		1.00	1.00
First generation		3.55 (3.48-3.62)	3.51 (3.44-3.58)
Second generation		2.04 (2.00-2.09)	2.02 (1.97-2.06)
Parenthood status			
Single parent		1.17 (1.14-1.19)	1.16 (1.14-1.19)
Two parents		1.00	1.00
Other		1.60 (1.50-1.71)	1.60 (1.50-1.70)
Parity			
Nulliparous		1.00	1.00
Multiparous		1.22 (1.20-1.24)	1.22 (1.20-1.24)
Maternal age			
<25		1.69 (1.65-1.73)	1.69 (1.65-1.73)
25-35		1.00	1.00
>35		0.98 (0.97-1.00)	0.98 (0.96-1.00)
**Neighbourhood level determinants**			
Urbanisation			
Urban			1.00
Moderate urban			0.79 (0.75-0.83)
Rural			0.73 (0.71-0.76)
Random effect			
Neighbourhood variance (SE)	0.62 (SE 0.01)	0.38 (SE 0.01)	0.35 (SE 0.01)
Proportional change in variance	Reference	0.39	0.44
Intraclass correlation	0.11	0.04	0.04

Abbreviations: OR: odds ratio, SE: standard error

*OR of 1.00 is meaning that the displayed category is the reference category

Table S9. Multilevel associations with individual determinants income, education, home ownership, migration background, parenthood household status, parity, and neighbourhood level determinants urbanisation and neighbourhood deprivation

	**Model 1:** Null model	**Model 2:** Individual level determinants added	**Model 3:** Neighbourhood level determinants added
Fixed effect, OR (95%-CI)			
**Individual level determinants**			
Disposable income			
Low <p20		1.74 (1.69-1.80)	1.75 (1.69-1.80)
Moderate p20-p80		1.12 (1.10-1.15)	1.13 (1.10-1.16)
High >p80		1.00*	1.00
Educational level			
Low		2.17 (2.08-2.26)	2.17 (2.08-2.27)
Intermediate		1.45 (1.42-1.48)	1.46 (1.43-1.49)
High		1.00	1.00
Home ownership			
Owner-occupiers		1.00	1.00
No-owner (renters/others)		1.68 (1.65-1.71)	1.66 (1.63-1.69)
Migration background			
Non-immigrant		1.00	1.00
First generation		3.55 (3.48-3.62)	3.50 (3.43-3.57)
Second generation		2.04 (2.00-2.09)	2.01 (1.96-2.05)
Parenthood status			
Single parent		1.17 (1.14-1.19)	1.16 (1.14-1.19)
Two parents		1.00	1.00
Other		1.60 (1.50-1.71)	1.59 (1.50-1.70)
Parity			
Nulliparous		1.00	1.00
Multiparous		1.22 (1.20-1.24)	1.22 (1.20-1.24)
Maternal age			
<25		1.69 (1.65-1.73)	1.69 (1.65-1.73)
25-35		1.00	1.00
>35		0.98 (0.97-1.00)	0.98 (0.96-1.00)
**Neighbourhood level determinants**			
Urbanisation			
Urban			1.00
Moderate urban			0.79 (0.76-0.83)
Rural			0.74 (0.71-0.77)
Neighbourhood deprivation			
No			1.00
Yes			1.12 (1.08-1.15)
Random effect			
Neighbourhood variance (SE)	0.62 (SE 0.01)	0.38 (SE 0.01)	0.35 (SE 0.01)
Proportional change in variance	Reference	0.39	0.44
Intraclass correlation	0.11	0.04	0.04

Abbreviations: OR: odds ratio, SE: standard error

*OR of 1.00 is meaning that the displayed category is the reference category

Supplementary file 3. Sensitivity analyses

Table S10. Sensitivity analyses with exclusion of consecutive pregnancies within the same mother

	**Model 1:** Null model	**Model 2:** Individual level determinants added	**Model 3:** Neighbourhood level determinants added
Fixed effect, OR (95%-CI)			
**Individual level determinants**			
Disposable income			
Low <p20		1.71 (1.65-1.76)	1.71 (1.66-1.77)
Moderate p20-p80		1.13 (1.10-1.15)	1.13 (1.10-1.16)
High >p80		1.00	1.00
Educational level			
Low		2.15 (2.06-2.24)	2.15 (2.06-2.25)
Intermediate		1.45 (1.42-1.49)	1.46 (1.43-1.49)
High		1.00	1.00
Home ownership			
Owner-occupiers		1.00	1.00
No-owner (renters/others)		1.66 (1.63-1.70)	1.65 (1.62-1.68)
Migration background			
Non-immigrant		1.00	1.00
First generation		3.52 (3.44-3.59)	3.46 (3.39-3.54)
Second generation		2.03 (1.98-2.08)	1.99 (1.95-2.04)
Parenthood status			
Single parent		1.17 (1.14-1.19)	1.16 (1.13-1.19)
Two parents		1.00	1.00
Other		1.60 (1.50-1.71)	1.59 (1.49-1.70)
Parity			
Nulliparous		1.00	1.00
Multiparous		1.26 (1.24-1.28)	1.71 (1.24-1.29)
Maternal age			
<25		1.71 (1.67-1.76)	1.71 (1.67-1.76)
25-35		1.00	1.00
>35		0.98 (0.96-1.00)	0.98 (0.96-1.00)
**Neighbourhood level determinants**			
Urbanisation			
Urban			1.00
Moderate urban			0.80 (0.76-0.84)
Rural			0.75 (0.72-0.78)
Neighbourhood deprivation			
No			1.00
Yes			1.11 (1.07-1.15)
Random effect			
Neighbourhood variance (SE)	0.60 (SE 0.01)	0.36 (SE 0.01)	0.34 (SE 0.01)
Proportional change in variance	Reference	0.40	0.43
Intraclass correlation	0.10	0.04	0.03

Abbreviations: OR: odds ratio, SE: standard error

*OR of 1.00 is meaning that the displayed category is the reference category

Table S11. Complete case analysis

	**Model 1:** Null model	**Model 2:** Individual level determinants added	**Model 3:** Neighbourhood level determinants added
Fixed effect, OR (95%-CI)			
**Individual level determinants**			
Disposable income			
Low <p20		1.68 (1.63-1.74)	1.69 (1.63-1.75)
Moderate p20-p80		1.11 (1.08-1.14)	1.12 (1.10-1.16)
High >p80		1.00	1.00
Educational level			
Low		2.75 (2.65-2.86)	2.73 (2.63-2.84)
Intermediate		1.58 (1.54-1.61)	1.58 (1.55-1.62)
High		1.00	1.00
Home ownership			
Owner-occupiers		1.00	1.00
No-owner (renters/others)		1.68 (1.64-1.71)	1.66 (1.62-1.69)
Migration background			
Non-immigrant		1.00	1.00
First generation		2.97 (2.90-3.05)	2.87 (2.80-2.95)
Second generation		1.97 (1.93-2.02)	1.92 (1.87-1.97)
Parenthood status			
Single parent		1.19 (1.16-1.22)	1.18 (1.16-1.22)
Two parents		1.00	1.00
Other		1.65 (1.53-1.78)	1.63 (1.50-1.76)
Parity			
Nulliparous		1.00	1.00
Multiparous		1.17 (1.15-1.19)	1.17 (1.15-1.20)
Maternal age			
<25		1.63 (1.59-1.68)	1.64 (1.59-1.68)
25-35		1.00	1.00
>35		1.01 (0.98-1.03)	1.00 (0.98-1.03)
**Neighbourhood level determinants**			
Urbanisation			
Urban			1.00
Moderate urban			0.77 (0.73-0.81)
Rural			0.70 (0.67-0.73)
Neighbourhood deprivation			
No			1.00
Yes			1.13 (1.09-1.17)
Random effect			
Neighbourhood variance (SE)	0.39 (SE 0.01)	0.16 (SE 0.01)	0.13 (SE 0.01)
Proportional change in variance	Reference	0.59	0.66
Intraclass correlation	0.11	0.05	0.04

Abbreviations: OR: odds ratio, SE: standard error

*OR of 1.00 is meaning that the displayed category is the reference category

Table S12. Sensitivity analyses with exclusion of women who did not receive any postpartum care

	**Model 1:** Null model	**Model 2:** Individual level determinants added	**Model 3:** Neighbourhood level determinants added
Fixed effect, OR (95%-CI)			
**Individual level determinants**			
Disposable income			
Low <p20		1.87 (1.81-1.93)	1.88 (1.82-1.95)
Moderate p20-p80		1.19 (1.16-1.23)	1.20 (1.17-1.23)
High >p80		1.00	1.00
Educational level			
Low		2.28 (2.19-2.37)	2.29 (2.20-2.38)
Intermediate		1.55 (1.51-1.58)	1.56 (1.52-1.59)
High		1.00	1.00
Home ownership			
Owner-occupiers		1.00	1.00
No-owner (renters/others)		1.78 (1.74-1.82)	1.76 (1.73-1.80)
Migration background			
Non-immigrant		1.00	1.00
First generation		4.15 (4.07-4.24)	4.08 (3.99-4.16)
Second generation		2.25 (2.19-2.30)	2.20 (2.14-2.25)
Parenthood status			
Single parent		1.17 (1.15-1.20)	1.17 (1.14-1.20)
Two parents		1.00	1.00
Other		1.61 (1.51-1.72)	1.60 (1.50-1.71)
Parity			
Nulliparous		1.00	1.00
Multiparous		1.19 (1.17-1.21)	1.19 (1.17-1.21)
Maternal age			
<25		1.73 (1.68-1.78)	1.73 (1.69-1.78)
25-35		1.00	1.00
>35		0.98 (0.96-1.00)	0.98 (0.96-1.00)
**Neighbourhood level determinants**			
Urbanisation			
Urban			1.00
Moderate urban			0.78 (0.74-0.82)
Rural			0.67 (0.65-0.70)
Neighbourhood deprivation			
No			1.00
Yes			1.10 (1.07-1.14)
Random effect			
Neighbourhood variance (SE)	0.67 (SE 0.01)	0.38 (SE 0.01)	0.34 (SE 0.01)
Proportional change in variance	Reference	0.43	0.49
Intraclass correlation	0.12	0.04	0.03

Abbreviations: OR: odds ratio, SE: standard error

*OR of 1.00 is meaning that the displayed category is the reference category
